# Spatial vitality variation in community parks and their influencing factors

**DOI:** 10.1371/journal.pone.0312941

**Published:** 2025-03-05

**Authors:** Jingjing Zhang, Xiao Hu, Juan Wang

**Affiliations:** 1 School of Civil Engineering and Architecture, Zhejiang Sci-Tech University, Hangzhou China; 2 College of Urban Construction, Zhejiang Shuren University, Hangzhou, China; Chitkara University, Punjab, INDIA

## Abstract

Community parks are the primary locations for urban residents to engage in nature, exercise, and establish social contact. This study used System for Observing Play and Recreation in Communities (SOPARC) to conduct field surveys of three typical community parks in Hangzhou, focusing on analyzing the differences in subspace vitality within community parks. First, we classified park spaces into four types: courts, lawns, fitness grounds, and pavilions. We selected 11 variables to assess the landscape features of the subspaces; then, visitor density, activity richness, activity evenness, and time stability were selected to quantify the vitality characteristics of the four space types. Finally, a mixed linear model was used to explore the relationship between landscape features and different types of spatial vitality and to select the key influencing factors affecting spatial vitality. The study found that (1) the vitality of different types of spaces in community parks differed significantly, showing three types of vitality. Among them, high activity richness and evenness, low visitor density and time stability were observed in courts and lawns. However, fitness grounds showed the opposite vitality pattern. Pavilions exhibited exceptionally high visitor density but low activity richness, activity evenness, and time stability. (2) The key influences affecting subspace vitality mainly include hydrophilicity and accessibility and exhibit enhancing or inhibiting effects on different types of spaces. The study found water-friendliness positively contributed to visitor density, activity richness, and time stability in all types of subspaces. Sports facilities increased visitor density and time stability on courts, lawns, and fitness grounds but reduced their activity evenness. Accessibility had an enhancing effect on activity richness but an inhibiting effect on the activity evenness of pavilions. We suggest that community parks be made more vibrant by adding natural elements to the space, installing multifunctional and compact facilities, and subdividing spatial functions.

## Introduction

As one of the fundamental types of green spaces in urban areas, community parks are the main carrying spaces for urban residents to get close to nature, relieve stress, exercise, and spend time with family and friends [1–3]. The high-frequency use of community parks not only improves the quality of life of urban residents but also enhances the social and economic vitality of the city, which is a crucial goal of community parks planning and management [4–6]. However, in the past, the design process of community parks was dominated by planners and policymakers’ aesthetic theory and users’ participation [7–9]. Unreasonable functional zoning and improper configuration of facilities have led to problems such as low utilization rates and a lack of vitality in community parks.

Currently, the term “spatial vitality” is widely used in the fields of urban design and sociology to denote the capacity and use of urban public spaces and the intensity of urban socioeconomic activities [10–12]. Several prominent researchers have emphasized the importance of creating vibrant public spaces. In her classic work, Jane Jacobs reflected on the impact of traditional urban planning on space utilization, suggesting that changes in spatial scale and quality levels have a critical impact on spatial vitality [13]. Gehl argued that improving the quality and vitality of public space in cities and neighborhoods requires focusing on the needs of residents for a variety of activity spaces [14]. Vitality-related research also covers a wide range of urban space scenarios such as parks [15–18], streets [19], and waterfront spaces [20]. These researchers have also underlined the importance of creating vibrant public spaces and the fact that a user needs perspective, which is key to assessing spatial vitality.

Research has focused on both external and internal factors that influence the vitality of park spaces. External factors include land use patterns, population density, and park accessibility around the park [21,22]. Land use patterns and population densities around the park would affect the efficiency of park use [23], Parks with better accessibility usually attract more tourists [24]. Internal factors involve park size and type, facility configuration [25], landscape type and quality [26], internal accessibility [27,28], and so on. For example, park size and type impact the visitations [29]. The configuration of sports facilities [30], recreational facilities [31], shelter and amenities [32,33] will significantly affect the duration and frequency of residents’ visits to the park. For example, a study in Zhengzhou, China, showed that in summer, shaded areas provided by trees or buildings usually attract more tourists and are used for more activities; high quality green landscapes and water features can bring more vitality to parks [10].

Overall, two research methods were employed to investigate park vitality. The first was a low-throughput survey method, which included field observations, questionnaire surveys, and interviews [34,35]. The other was the big data analysis method based on mobile phone signaling, GPS, LBS, and social media data [36,37]. With the rapid development of big data, many data collection and visualization methods have been designed, including those applicable to urban public space research. Multisource big data analysis has advantages such as large data volume and easy accessibility. It is also more suitable for comparing multiple large-scale urban public spaces with a broader scope [38,39]. However, its disadvantages include limited population coverage and low data accuracy, making it difficult to translate it into research on small public spaces [40]. Gathering data through on-site observations is a more precise way of capturing information, including visitor counts and behavior, which are crucial in comprehending how people utilize a particular space.System for Observing Play and Recreation in Communities (SOPARC) is a non-participant, momentary batch sampling-based physical activity scale proposed by Mckenzie [41]. SOPARC can better capture the behavioral characteristics of visitors and has been widely used in the field of urban open space research [42,43].

However, most studies on the park spatial vitality have looked at the parks as a homogeneous space. Only a few studies have focused on spatial heterogeneity within community parks [44,45]. Nevertheless, the distribution of tourists in community parks is extremely uneven and the internal spaces of community parks are highly spatially heterogeneous [46]. From the perspective of material composition, the land space of most community parks can be divided into four types: square spaces mainly paved with hard materials and with a large area, semi-indoor spaces composed of pavilions and corridors for activities, spaces with centralized fitness configurations, and lawns providing natural space. Tourists use these spaces in different ways, including activity types, frequency of use, duration of stay, and so on [47], resulting in different levels of spatial vitality. Our aim is to carefully examine the vitality differences within the subspaces of the park, and based on this, identify and analyze the key influencing factors that affect the vitality of each subspace. This can not only enhance the overall vitality of the community park but also ensure that various subspaces within the park are fully utilized, thereby improving park efficiency.

In this study, we classified community park spaces into four subspace types: courts, lawns, fitness grounds, and pavilions. Three community parks in Hangzhou, China, were selected to assess spatial vitality from four dimensions, namely, visitor density, activity richness, activity evenness, and time stability. We then analyzed the vitality characteristics of different types of spaces, detected the relationship between landscape features and the vitality of different subspaces, and determined the mechanism of their influence, thereby providing insight into the planning and design of parks to improve the overall vitality of community parks.

## Materials and methods

### Study area

Located in the northern part of the southeast coast of China (118°21′E-120°30E, 29°11′N-30°33N′), Hangzhou is the capital of Zhejiang Province. By the end of 2022, the central urban area of Hangzhou covered 1,010.3 km^2^, the total population of the urban area was 12.376 million, and the rate of the green areas coverage in constructed areas was 39.74% (Hangzhou Bureau of Statistics).

**Fig 1:**
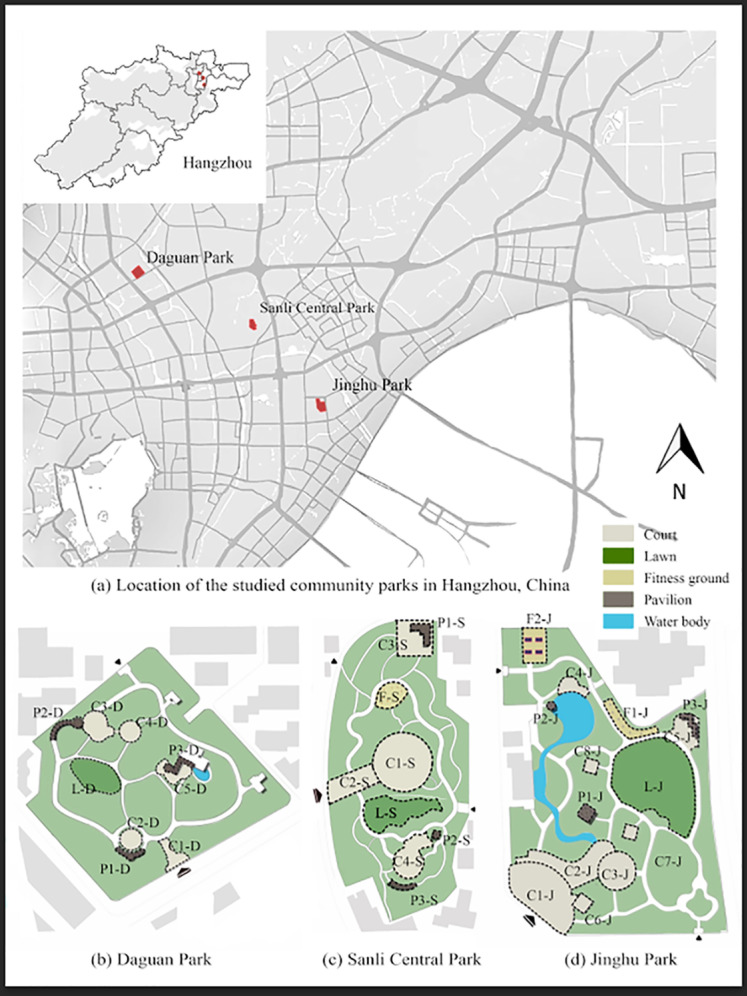
Location and Spatial Arrangement of the Three Studied Community Parks. **(a)** Location of the Three Parks in Hangzhou; **(b)** Subspace Arrangement of Daguan Park; **(c)** Subspace Arrangement of Sanli Central Park; **(d)** Subspace Arrangement of Jinghu Park. Source: Created by the author based on the base map of Hangzhou from the Open Street. Map (OSM) geographic data platform (https://www.openstreetmap.org/).

Employing the research framework of in-depth analysis of parks’ internal subspaces, as a case study, three community parks were carefully chosen to ensure the relative consistency of the external environment while highlighting the diversity of the parks’ internal spaces and landscape elements. We focused on the densely populated areas to the northeast of Hangzhou city and selected three community parks with similar transportation conditions and population densities: Daguan, Sanli Central, and Jinghu Parks (Fig 1, Table 1). These three community parks are all located in the most densely populated areas of Hangzhou and are surrounded by residential land with good transportation conditions, high accessibility, and a sufficient sample of visitors. In addition, parks are rich in landscape features, making it easy to analyze the relationship between spatial vitality and landscape features.

**Table 1:** Description of the studied community parks.

	Daguan Park	Sanli Central Park	Jinghu Park
District	Gongshu District	Shangcheng District	Shangcheng District
Total area (ha)	1.48	1.00	1.53
Green Area proportion (%)	82.3	70.5	68.7
Water Area proportion (%)	0.70	0.00	3.01
Number of entrances and exits	2	3	3
Facilities available in the park	1, 5, 6, 7, 8, 9, 10	1, 2, 3, 5, 6, 7, 8, 9, 10	1, 2, 4, 5, 6, 7, 8, 9, 10

1 = walking/cycling paths, 2 = fitness equipment, 3 = children’s playground, 4 = Table tennis tables, 5 = multipurpose open space, 6 = multipurpose sheltered space, 7 = Lighting, 8 = Seating, 9 = Toilet, 10 = Sculptures.

### Data collection

#### Landscape features of studied subspaces.

In this study, community park spaces were classified into four subspace types: courts, lawns, fitness grounds, and pavilions (Table 2). Three community parks were divided into 32 subspaces and numbered accordingly. The final number comprised the subspace type, number, and Chinese acronym of each community park. As an example, P1-D refers to Pavilion 1 in Daguan Park (Fig 1).

**Table 2:** Characteristics of the subspace types in community parks.

Type	Shape	Area (m^2^)	Pavement	Description
Court	Rectangular or round	64 ~ 846	Impervious surface	Open spaces enclosed by paths and vegetation, which can be divided into several local spaces, equipped with seats, activity facilities and landscape sketches
Lawn	Irregular	450 ~ 828	Grass	Large open spaces surrounded by paths and vegetation, slightly undulating
Fitness ground	Irregular	169-217	Plastic	Open spaces that compactly arranged the fitness facilities, such as outdoor fitness equipment (OFE), table tennis table and basketball court
Pavilion	Dotted or banded	15 ~ 103	Impervious surface	Formed by landscape architecture such as pavilions, corridors, and trellises, with a roof but no walls, with ample seating and shelter facilities

Following previous studies [38,48], we measured the landscape features of the subspaces using 11 distinct variables: area, PAR, obscuration, openness, seating facility, sports facility, green view ratio, landscape sketch, water-friendliness, accessibility, and connectivity, among which eight variables characterized the landscape features of the subspace itself (area and shape, degree of enclosure, and presence of facilities) and three variables measured the surrounding environment of the subspace (water-friendliness, connectivity with paths, and other subspaces). These variables were used to assess the association between landscape features and spatial vitality. Table 3 shows a detailed description and calculation methods for these variables.

**Table 3:** Landscape Features of the Studied Subspaces.

Landscape features	Unit	Description and calculation method
Area	m^2^	Area of the subspace
PAR	m/m^2^	PAR = perimeter/area. The larger value means the more irregular shape
Obscuration	—	Enclosure in the vertical orientation We chose four corners (east, south, west, and north) of each sample subspace as a photographic point and took four photos of the sky from there at the height of human perspective (1.5 m) $O=\frac{\sum_{i=1}^{4}Area_{sky}}{\sum_{i=1}^{4}Area_{total}}\times$ 100% O: Obscuration, *Area*_*sky*_: area of the sky, *Area*_*total*_: area of the total image *i*: Number of images collected at the observation point O ≤ 10%:1; 10% < O ≤ 40%:2; 40% < O ≤ 70%:3; O > 70%:4
Openness	—	Openness in the horizontal orientation Single-sided opening:1; two-sided opening: 2; three-sided opening:3; full opening: 4
Seating facility	—	The ratio of the length of the seating facilities of the boundary. The larger the value, the more seats Sf = 0:0; 0 < Sf ≤ 0.1:1; 0.1 < Sf ≤ 0.3:2; Sf > 0.3:3
Sports facility	Number	Number of sports facilities, such as outdoor fitness equipment, table tennis table, and basketball court
Green view ratio	%	Proportion of green elements in the total view field We chose the center of each sample space as a photographic point and took one picture from there to the east, south, west and north respectively of the surrounding greenery at the height of human perspective (1.5 m) $D_{g}=\frac{\sum_{i=1}^{4}Area_{green}}{\sum_{i=1}^{4}Area_{total}}\times$ 100% D_g_: Green view ratio, *Area*_*green*_: area of the greenery, *Area*_*total*_: area of the total image, *i*: Number of images collected at the observation point
Landscape sketch	Number	Number of landscape sketches, such as monuments, statues, steles, and fountains
Water-friendliness		Visitors’ proximity to water in the sample space Accessible to water (pond. lake):3; Visible but inaccessible: 2; Invisible and inaccessible: 1
Accessibility	m	Distance to the nearest entrance
Connectivity	m	Distance to the nearest subspace

### Observations of visitors

In this study, we used SOPARC to collect information on the number, sex (male and female), age (older adults [ > 60], middle-aged adults [40–60], young people [16–40], and children [0–16]), and behaviors of visitors in 32 subspaces of three parks.

Formal research was conducted from September to October 2022, when Hangzhou had a comfortable climate for outdoor activities. Three weekdays and three weekends with sunny weather were selected, and 15 time periods were recorded hourly from 7:00 am to 9:00 pm. A total of 14,530 visitor samples were obtained: 4,184 from Daguan Park, 4,840 from Sanli Central Park, and 5,506 from Jinghu Park. A total of 23 types of activities were recorded during the on-site research.

### Evaluation of spatial vitality

According to the vitality representation factors proposed by Gehl, how many people use the spaces, how long their activities last, which activity types can develop, and the diversity of these activities are crucial for the creation of vitality [14]. Both classical and recent studies on open space vitality indicate that it is more meaningful and reasonable to quantify vitality as a multifaceted indicator, especially in small public spaces [13,49,50]. Generally, vitality comprises the following elements: number of people, activity duration, activity diversity, and activity type. Based on previous studies, we assessed the spatial vitality of different subspaces in four dimensions: visitor density, activity richness, activity evenness, and time stability.

Visitor density (AD) refers to the ratio of the number of visitors to the area of a sample subspace and is used to measure the intensity of the use of the space. The larger the value, the stronger the vitality.

Activity richness (AR) refers to the number of observed activity types in a sample subspace. The higher the value, the more types of activities a sample subspace can support.

Activity evenness (AE) is quantified using Shannon’s Evenness Index. Higher values indicate a higher evenness of individual numbers among different types of activities; in other words, lower activity preference. The formula is as follows:


$$\text{AE}=-\overset{S}{\underset{i=1}{\sum }}\left[P_{i}\text{*}\log P_{i}\right]/\text{ln}\left(S\right)$$

$$P_{i}={\text{m}}_{i}/M$$

where *P*_*i*_ is the proportion of the *i*th activity type, *m*_*i*_ is the number of visitors in the sample subspace who performed the *i*th type of activity, *M* is the total number of visitors in the subspace, and *S* is the number of activity types in the sample subspace.

Time stability (TS) was the inverse of the coefficient of variation in the visitor number across time. It is used to reflect changes in the density of active populations over time. The higher the value, the longer the subspace is at an active level and more efficiently utilized. The formula is as follows:


$$TS=MEAN\left(visitornumberacrosstime\right)/SD\left(visitornumberacrosstime\right)\times 100\%$$

### Statistical analysis

After normalizing the landscape feature data, the vitality of the four subspace types was measured based on the four indicators mentioned above. The natural discontinuity method was used to divide the results into five levels and obtain vitality analysis graphs for each subspace. A one-way analysis of variance (ANOVA) was used to test the vitality variation across the four types of subspaces. We then characterized and compared the overall vitality characteristics of the four types of subspaces in the radar charts. Furthermore, a mixed linear model was employed to explore the effects of landscape features on the spatial vitality of open spaces and pavilions separately (see Table 3). All 11 landscape features were selected as fixed-effects factors for the open space model, and nine landscape features were selected as fixed-effects factors for the pavilion model (with the removal of sports facilities and landscape sketches). Park identity and subspace type were controlled for as random effects.

## Results

### Vitality characteristics of different subspaces

**Fig 2:**
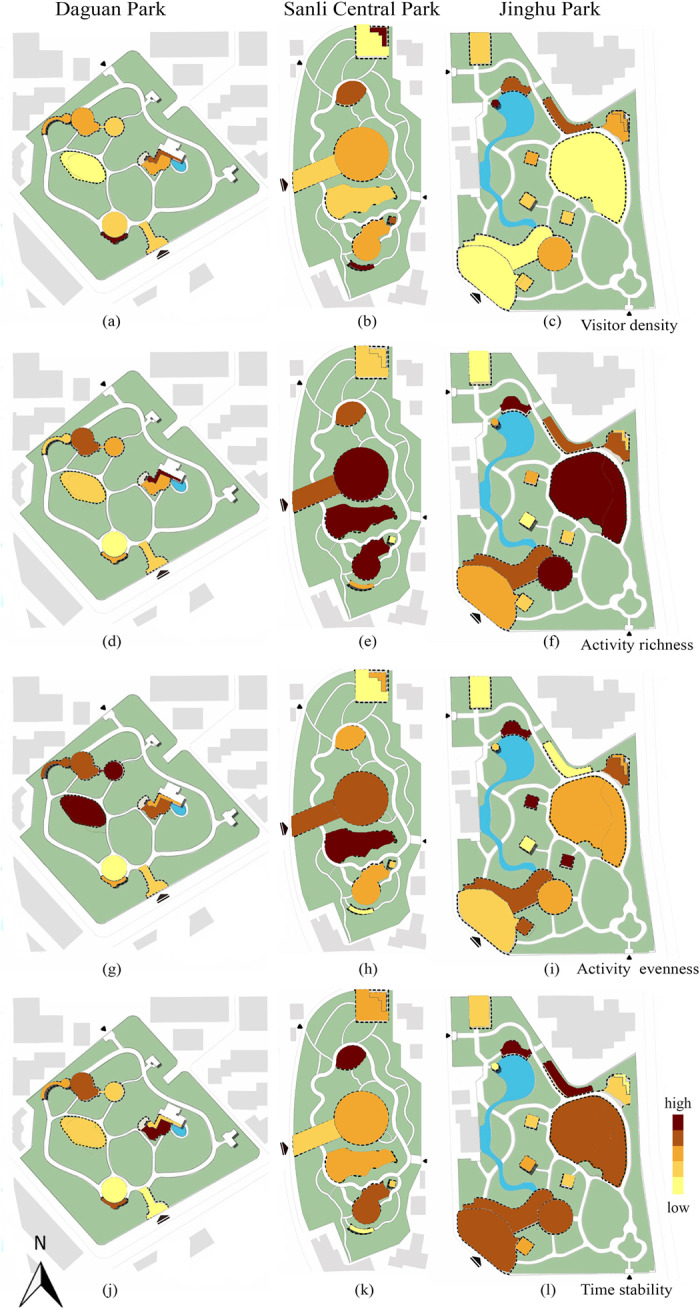
Distribution map of vitality in each subspace of the sample parks.

**Fig 3:**
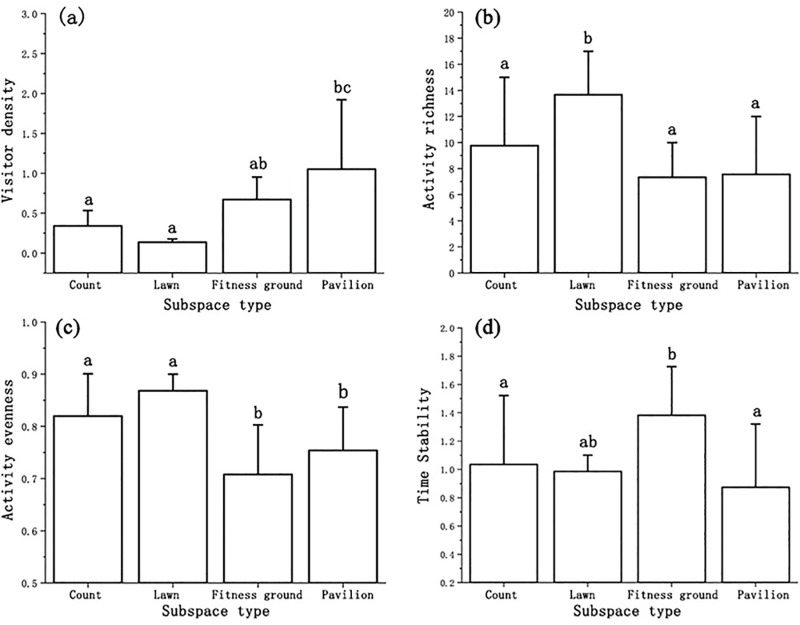
Four Vitality Characteristics of Different Subspace. **(A)** Visitor Density; **(B)** Activity Richness; **(C)** Activity Evenness; **(D)** Time Stability.

The vitality of different types of subspaces exhibited strong spatial heterogeneity (Fig 2, Table 4, Fig 3). In terms of visitor density, pavilions performed the best (Fig 2), with an average of 1.0519 p/m2. Moreover, they differed significantly from courts and lawns, which had a low visitor density, with average values of 0.3411 p/m2 and 0.1367 p/m2, respectively. The fitness grounds exhibited intermediate density levels. The richness of activities on the lawns was significantly higher than that in the other three spaces, with an average of 13.6777; there were fewer types of activities on the fitness grounds and in the pavilions, with average values of 7.3333 and 7.5556. Lawns and courts performed better in terms of activity evenness, with average values of 0.8682 and 0.8198. They were significantly different from the fitness grounds and pavilions. In terms of time stability, the fitness grounds performed the best, with an average of 1.3827, followed by courts, lawns, and pavilions. There was a marked difference between lawns and courts, with the same low stability.

**Table 4:** The average vitality index values for the different subspace types.

	Visitor density (p/ha)	Activity richness	Activity evenness	Time Stability
Court	0.3411	9.7647	0.8198	1.0349
Lawn	0.1367	13.6667	0.8682	0.9855
Fitness ground	0.6720	7.3333	0.7079	1.3827
Pavilion	1.0519	7.5556	0.7540	0.8735

**Fig 4:**
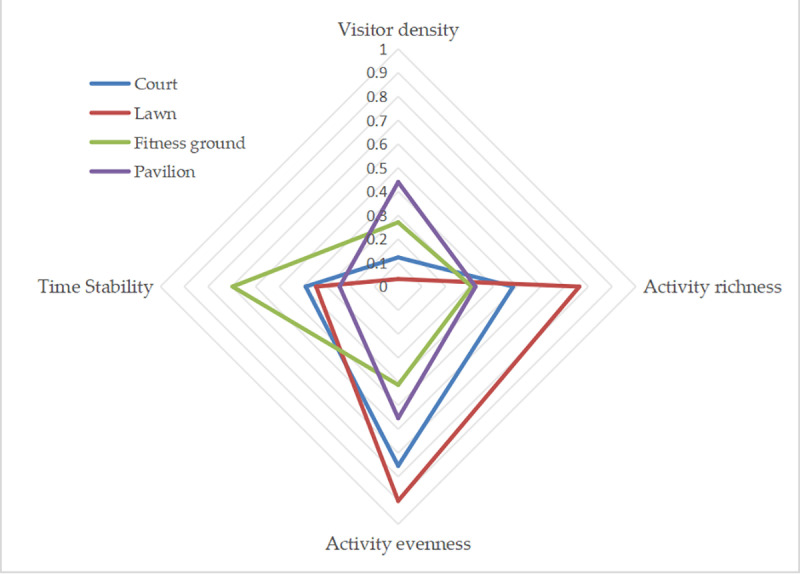
Comparison of Vitality Indicators Between Four Subspace Types.

To compare the differences in the vitality characteristics of different subspaces, the study used the normalization method to unify the dimensions of the average values of the four vitality indicators and drew a radar chart of the vitality indicators (Fig 4). Research has identified three vitality characteristics among the four types of subspaces in community parks. Lawns and courts had high activity richness and evenness; however, visitor density and time stability were low. The vitality characteristics of fitness were the opposite, with higher visitor density and time stability and lower activity diversity and evenness. Pavilions had an exceptionally high visitor density and low activity richness, evenness, and time stability.

### Effects of landscape features on spatial vitality

In the group of open space, time stability, visitor density, and activity richness were significantly influenced by landscape features. Water-friendliness and sports facilities not only increased time stability but also contributed to visitor density. High accessibility increased the time stability of the space, but the larger the area, the lower the visitor density. Landscape sketches, water-friendliness, and accessibility contributed to the activity richness of open spaces. Surprisingly, sports facilities were negatively correlated with activity evenness (Table 5).

**Table 5:** Effects of Landscape Features on Spatial Vitality by Mixed Linear Models Controlling the Influence of Park Identity and Subspace Type.

Spatial vitality	Factor^*^	Estimated Coefficient	Sum Square	*F*-Value	*P*-Value
Open space					
Visitor density (*R*^2^ = 60.6%)	Water-friendliness	0.12	0.29	11.74	0.003
	Sports facility	0.1	0.152	8.36	0.023
	Area	-0.08	0.14	4.7	0.045
Activity Richness (*R*^2^ = 54.1%)	Landscape sketch	1.68	47.18	7.89	0.011
	Water-friendliness	1.33	37.37	6.25	0.022
	Accessibility	1.33	20.32	5.07	0.036
Activity evenness (R^2^ = 17.4%)	Sports facility	-0.04	0.029	3.94	0.09
Time Stability (*R*^2^ = 64.6%)	Sports facility	0.08	0.153	15.53	<0.001
	Water-friendliness	0.08	0.153	15.48	<0.001
	Accessibility	0.05	0.054	5.48	0.03
Pavilion^**^					
Visitor Density (*R*^2^ = 50.8%)	Water-friendliness	0.24	0.408	10.08	0.018
Activity Richness (*R*^2^ = 18.2%)	Water-friendliness	1.17	7.263	16.01	0.016
	Accessibility	1.11	5.832	12.85	0.023
Activity evenness (*R*^2^ = 83.8%)	Connectivity	0.09	0.007	8213312	<0.001
	Green view ratio	0.06	0.006	6573500	<0.001
	Area	0.04	0.002	2289602	0.002
	Accessibility	-0.05	0.004	4903944	0.001

*Only significant factors were listed. **The time stability of pavilions was insignificantly affected by the landscape features, which was not listed in the table.

In the pavilion group, activity evenness was extremely sensitive to changes in landscape features, and the landscape features that contributed significantly were connectivity and green view ratio, followed by area. In contrast, accessibility had an inhibitory effect on it. Visitor density and activity richness were also influenced to some extent by landscape features, with water-friendliness increasing both the density and activity richness of spatial vitality and accessibility contributing to richness. In addition, none of the variables significantly affected time stability (Table 5).

## Discussion

### Spatial vitality variation of subspaces

#### Spatial vitality of courts and lawns.

Courts and lawns are common open spaces in community parks. They share the vitality characteristics of richer activity types, fewer visitors, activity preferences, and efficient temporal utilization. Most of these subspaces are more accessible and open and are generally equipped with seating facilities and landscape sketches, fulfilling the demands of visitors’ outdoor activities. Group exercises such as dancing, Tai Chi, and chorus, as well as walking, riding, and skateboarding, require large spaces and, thus, usually occur in courts and lawns. Landscape sketches also attract many children to spaces for activities such as climbing and chasing. In addition, small spaces divided by plants and sketches have more people sitting, chatting, looking at cell phones, and being involved in other resting and socializing activities. However, perhaps because of their large size, visitor density is relatively low in courts and lawns.

Compared to courts, lawns have a wider variety of activity types and a greater evenness of activity. There seems to be a consensus that lawns are a popular recreational space in Western Countries [51,52], whereas in China, due to restricted access, lawns exist as an ornamental landscape, and therefore, the spatial vitality is not high [53]. However, 17 different types of activities were found on the lawns of Jinghu Park, the most of any of the sample subspaces. The natural substrate of the lawn induces many nature-based educational activities, such as catching bugs and collecting leaves, as well as soccer and camping, which are more challenging to carry out in other subspaces. Courts, however, are generally hard-paved with a flat surface that is more in line with the needs of people engaged in square dancing, riding, skateboarding, and other activities.

In addition, the peak hours and usage intensities of the two types of subspaces differed. Due to congregate fitness activities, such as dancing and calisthenics, the number of visitors to the courts peaks at 7-9 am and 6-8 pm. Meanwhile, lawns reach their usage peak only once a day, at 4-6 pm after school and before dinner, with a large number of people playing football, and are less heavily utilized for the rest of the day.

#### Spatial vitality of fitness grounds.

Fitness grounds exhibit diametrically opposite vitality characteristics to courts and lawns. It is the subspace with the highest visitor density and time stability of community parks but with low activity richness and evenness. This type of subspace has distinct boundaries and is equipped with outdoor fitness facilities such as OFE, ping pong tables, and basketball areas for visitors to work out. Fitness grounds in community parks have become the preferred places for outdoor fitness activities for many residents because of their proximity to their homes, low cost, simple equipment operation, and flexibility of usage [54]. On-site observations revealed that regardless of whether it was a weekday or weekend, the fitness grounds in the sample parks had a large number of visitors during hours other than 11 am-1 pm. In addition, ping pong is a popular sport in China, and the table tennis tables are also used efficiently. However, the fitness ground area is generally small, fitness facilities are densely distributed, and the function of the facility limits the type of activity in this space, resulting in low activity richness and evenness. People visit this space with clear purposes, focusing on equipment fitness, ball games, and a small number of children’s activities.

#### Spatial vitality of pavilions.

Pavilions have vitality characteristics such as high visitor density, single activity types, obvious preferences, and concentrated usage time. This type of subspace has a high level of enclosure, with sufficient tables, chairs, and shading facilities; however, the area is small, resulting in a high density of visitors. This also limits most outdoor activities and is only strongly attractive for sitting, chatting, chess, and other similar activities. At the same time, these activities exclude those with high intensity and noise, affecting the evenness of spatial vitality. Based on on-site observations, pavilions located far from the main paths have greater privacy and are more prone to activities such as sitting and chatting. Pavilions or corridors located near main paths or conveniently accessible areas tend to attract activities such as playing cards and chess. In addition, the time for efficient utilization is relatively short, with peak usage occurring at 2-4 pm due to group chess and card activities.

### Roles of landscape features in spatial vitality

#### Hydrophilic spaces have higher spatial vitality.

Whether it is an open space or a garden architectural space, hydrophilicity has a positive promoting effect on increasing the visitor density, richness of activity types, and time stability of the space. This is consistent with previous findings [55]. A beautiful water feature not only improves the ornamental nature of the space but also enhances the visitor’s visiting experience, thus triggering more activities to occur at more times. Water is particularly attractive for children [56]. It was observed in the field that children utilized water bodies for activities such as catching bugs, fishing, and splashing. In the meantime, their parents or caregivers tended to rest, talk, or engage in physical exercise in adjacent spaces.

#### The trade-off of fitness facilities for spatial vitality in open spaces.

In the open space group, we found that sports facilities contributed to visitor density and temporal stability but inhibited activity evenness. Due to the COVID-19 pandemic, some attributes of outdoor activity have become ever more critical, and the need for outdoor physical activity has dramatically increased. However, the more facilities in spaces, the greater the likelihood of people engaging in fitness activities, whereas the supportability for other activities decreases, especially in spaces with dense activity facilities and fewer facilities for rest or communication.

#### The trade-off of accessibility for spatial vitality in pavilions.

In the pavilion group, accessibility also shows a trade-off effect, which increases the activity richness of the space but has a dampening effect on evenness. High accessibility, which increases the likelihood of visitors traversing the space, does not necessarily result in a long-term stay and does not represent the typical activity characteristics of the space. In particular, in pavilions that have a pass-through function, it is easy to trigger pass-through activities such as walking and jogging. These activities can easily interfere with the original activities in the space [57]. The smaller the area and narrower the shape, the more evident the inhibition.

### Implications

First, it is highly recommended to add spaces with natural elements, such as water bodies and lawns, to parks, as they can effectively increase the intensity and richness of space use. The form of water bodies can be modeled after meandering streams in their natural state, increasing the interface between land and water, thus enhancing the attractiveness of the space, provided that safety and hygiene are met. Water-friendly platforms and walkways can be erected to enhance the possibility of visitors coming into direct contact with water. In addition, a larger number of small, scattered lawns in a community park can be more conducive to activities than a single large lawn. The lawn can also be furnished with vignettes.

Second, considering the promotion effect of activity facilities on visitor density and time steadiness, as well as the inhibition effect on activity evenness, it is necessary to rationally arrange their types, quantities, and locations. In fitness grounds that have a high intensity of use and time utilization but are singularly active, designers should optimize the type and number of facilities from the perspective of the functional composite. At the same time, at the edge of the space or adjacent space, supplemental rest, landscape, shade, and other facilities can be added to meet the possible needs of visitors after fitness rest and foster communication. Courts and lawns can be arranged with more functional composite, movable, simple activity facilities, such as a half-court basketball court and tennis rackets, or combined with landscape sketches to provide some passable, climbing, and sliding facilities to meet the needs of visitors of different ages. Facilities are appropriately located around the perimeter of the space to avoid conflict with other activities in the space.

Third, spatial functions should be clear and refined. Reasonable functional zoning can reduce the conflict between different behaviors due to ambiguous functional positioning, thus allowing space to accommodate more activities at more times and attract more visitors. Especially in large-scale open spaces, such as courts and lawns, the differences in the active population, activity time, and demand for space, facilities, and landscapes for different activities should be carefully studied. Plants, water bodies, terrain, steps, and other tactics could be utilized for spatial division and integration.

### Contributions and limitations

#### Contributions.

Community parks are important places for the fitness, leisure, and socialization of urban residents. Understanding the heterogeneity of spatial vitality within community parks and its relationship with the characteristics of spatial elements is of practical significance for improving the effectiveness of park use and the quality of life of residents. The cases in this study are three community parks in Hangzhou. Located in the Jiangnan region of China, Hangzhou is known for its excellent ecological environment and livability, and its community parks have achieved remarkable results in building a high-quality and efficient park system. Given that Hangzhou is deepening the process of urban park construction, the results of this study not only provide empirical evidence for current and future park layout optimization and planning design but also help to reconcile the synergistic relationship between planning policies and urban design strategies. These results can be used to achieve the goals of efficient allocation of resources and sustainable development of the city. In addition, the evaluation and analysis methods used in this study are generalizable and are expected to provide a reference for the evaluation of park performances in other regions and countries.

This study also contributes to the literature on park construction. First, the study validates the theory of place–human interaction and discusses the constraints and influences of urban space on behavior. Using three community parks in Hangzhou as empirical evidence, we analyzed the differences in vitality levels of four park subspaces through one-way ANOVA and radar charts and explored the effects of different landscape features on crowd activities through mixed linear models. We found that different landscape features influence the behaviors of the crowd within them in different ways; furthermore, there are trade-off effects of activity facilities and accessibility on different indicators of the site’s crowd activity characteristics. This finding deepens our understanding of the relationship between urban space and behavior. Second, the study enriches the analytical dimension of park spatial vitality by drawing from the comparison of subspaces within parks to identify and analyze differences in the vitality of subspaces within urban community parks. This innovative perspective not only enriches the theoretical level of park spatial vitality research but also provides a more refined analytical framework for subsequent research, which helps to explain the multidimensional characteristics of spatial vitality and its influencing factors in a more comprehensive way. Thus, the results provide scientific guidance for the design and management of community parks.

### Limitations and future research opportunities

It is worth noting that this study has some limitations. First, the study suffers from the limitations of a relatively small survey size and a small sample park. The method used to obtain the data was an on-site survey, which was time-consuming and required more human, material, and financial resources than the collection of big data; this, to a certain extent, limited the broad applicability and generalizability of the study’s conclusions. In future research, we will expand the geographical scope of the study and select more diverse park spaces for long-term, systematic on-site observation to more comprehensively capture the activity needs of different groups of people and the impact of site landscape features on their activity patterns.

In addition, as data from different sources may produce discrepancies or even controversial conclusions, we must maintain a cautious attitude in the data analysis process and seek mutual verification and supplementation of data from multiple sources. In order to enhance the objectivity and scientificity of the study, future research will endeavor to build a diversified data collection system that not only includes traditional field survey data but also introduces mobile data and other big data sources. Through the comprehensive comparison and fusion analysis of big data and field survey sample data, we will be able to more accurately grasp the actual face of the spatial vitality of community parks and provide more solid data support for urban planning and park design.

## Conclusions

In this study, two evaluation systems were constructed. One of the systems selected 11 variables to measure the landscape features of the subspaces in parks, and the other constructed an indicator system to evaluate the vitality of parks from four dimensions: user density, activity diversity, activity uniformity, and time stability. Taking three community parks in Hangzhou as typical cases, the study used these evaluation systems to comparatively analyze the subspace characteristics of community parks. It was found that there were distinct differences in the vitality of different types of spaces in community parks, lawns, and courts that possess higher activity richness and evenness but lower visitor density and time stability; the vitality characteristics of fitness grounds are opposite to them, and the space of landscape architecture possesses particularly high visitor density and lower activity richness, evenness, and time stability.

The study further employed mixed linear modeling to explore the relationship between landscape features and different types of spatial vitality. The analysis shows that the effects of landscape features on the vitality of different spaces show synergies and trade-offs. The former, such as hydrophilicity, positively contributes to different dimensions of vitality in community park spaces. Although sports facilities increase the visitor density and temporal stability of open spaces reduce the preference for spatial vitality, accessibility promotes the richness and temporal stability of spaces but inhibits the activity and evenness of landscape architecture spaces.

In summary, this study focuses on the subspaces within community parks, systematically measuring their vitality and successfully revealing the relationship between site landscape features and spatial vitality. Our empirical study shows that there are synergistic and trade-off effects of park landscape features on crowd activities. These findings provide not only effective data information for the design and management of community parks but also valuable references for future park construction in other areas. We expect that these findings will be widely used in practice to promote the development of community park design in a more humanized and scientific way.

## Supporting information

S1 Data Dataset on the landscape features of the studied subspaces. (XLSX)
